# Transmission Electron Microscopy for Structural Insights into Bacterial Cellulose Nanowhiskers in Ternary Deep Eutectic Solvent

**DOI:** 10.1021/acsmeasuresciau.5c00159

**Published:** 2026-01-02

**Authors:** Maurelio Cabo, Abed Alqader Ibrahim, Gayani Pathiraja, Farbod Ebrahimi, Shobha Mantripragada, Omiya Ayoub, Besan Khader, Kristen Dellinger, Jeffrey R. Alston, Sherine O. Obare, Dennis R. LaJeunesse

**Affiliations:** † Department of Nanoscience, Joint School of Nanoscience and Nanoengineering, 14616 The University of North Carolina at Greensboro, Greensboro, North Carolina 27401, United States; ‡ Department of Nanoengineering, Joint School of Nanoscience and Nanoengineering, 3616 North Carolina Agricultural and Technical State University, Greensboro, North Carolina 27402, United States

**Keywords:** Bacterial cellulose, Nanowhisker, Ternary Deep Eutectic Solvent, Spectroscopy, TEM, Thermal Stability, Thermal Analysis

## Abstract

In this study, we used Transmission Electron Microscopy (TEM) to establish that bacterial cellulose formed nanowhisker (BCWN) rod-like structures with distinct short and long morphologies when fabricated in a ternary deep eutectic solvent (TDES) of choline chloride, imidazole, and tannic acid. STEM-EDS confirmed nanowhisker twisting, diameter, elongation, and elemental composition. BCNWs enhanced TDES thermal stability by increasing the decomposition temperature and residual yield. DLS and zeta potential showed particle size enlargement and charge reversal, while DSC indicated a reduced melting temperature and restricted molecular mobility. Thus, TEM not only elucidated cellulose morphology but also provided insights into structural transformations and the reinforcing role of BCNWs in tuning eutectic solvent properties for sustainable nanomaterials and potential polymer electrolyte applications.

## Graphical abstract

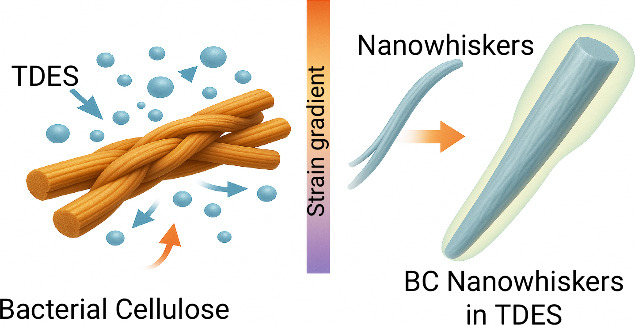

Bacterial cellulose nanowhiskers (BCNWs) are highly crystalline structures valued for their low density, biodegradability, and mechanical strength as reinforcements in nanocomposites.^1^ Conventional production methods often depend on harsh acids or energy-intensive treatments, conflicting with green-chemistry goals.^2^ Deep eutectic solvents (DESs) offer a sustainable alternative due to their tunable properties, low toxicity, and environmental compatibility.^3,4^ Ternary DESs (TDESs) expand solvent versatility and have been applied in biomass dissolution, extraction, and polymer stabilization.^5,6^ However, the structural characteristics of biomass after TDES-based degradation remain insufficiently studied. Here, we report BCNWs fabricated in a choline chloride–imidazole–tannic acid TDES, assessing diameter, length, elongation, twist, particle size, and zeta potential, as well as their effects on TDES thermal stability and kinetics. This TDES was chosen because choline chloride has low toxicity,^7^ imidazole forms strong C2–H···O hydrogen bonds with cellulose,^8^ and tannic acid enhances hydrogen-bond restructuring to act as a cosolvent.^9^ Meanwhile, TEM enables precise structural characterization,^10^ and understanding these features is important, as tuning bacterial cellulose (BC) structure can improve nanocomposite performance, stabilize green solvents, support biodegradable films, and enable liquid-crystalline or optical functions for sustainable material applications.

To confirm the transformation of bacterial cellulose into nanowhiskers, we revisited and repeated our previous study of solubilizing bacterial nanocellulose into ternary deep eutectic solvent wherein five successful runs were observed, see Table S1 and S2,^6^ and Table S3 discloses its length and diameter. Here, we are interested in further characterizing the runs that produced the shortest and longest nanowhiskers. For easy understanding, we renamed E1 as TDES/BCNW_S and E5 as TDES/BCNW_L. To understand how the transformation occurs, in Figure 1A, the structural features of BC prior to dissolution, BC_OD for TDES/BCNW_S and BC_MD for TDES/BCNW_L, were characterized by using atomic force microscopy (AFM) and scanning electron microscopy (SEM). AFM images revealed distinct surface textures, with oven-dried BC exhibiting a mean roughness (*S*_a_) of 26.65 nm and maximum pit depth (*S*_*y*_) of 128.30 nm, Figure 1A­(i), while microwave-dried BC showed slightly higher roughness (30.00 nm) and pit depth (132.70 nm), Figure 1A­(ii). SEM analysis confirmed a dense fibrous network with an average fibril diameter of 45.03 nm for BC_OD, Figure 1A­(iii), and 45.57 nm for BC_MD, Figure 1A­(iv). Upon exposure to the TDES system, the BC network was disrupted, enabling fibrillar disintegration. To evaluate the nanowhiskers in TDES, SEM imaging was first attempted, Figure 1B. While SEM provided some indication of whisker-like features, the images lacked sufficient contrast and resolution to resolve nanoscale details in the liquid environment, limiting reliable measurement. In comparison, transmission electron microscopy (TEM), Figure 1C, proved far more effective, offering high-resolution visualization of nanowhiskers suspended within the solvent matrix. TEM clearly revealed rod-like whiskers with measurable variations in morphology between different TDES formulations. In Figure 1D, TDES/BCNW_S TEM analysis showed short nanowhiskers with diameters of 5.94 ± 1.84 nm and lengths of 174.33 ± 8.4 nm, reflecting significant fibril exfoliation and thinning and an 87.5% reduction in diameter relative to the original BC fibrils. In contrast, TDES/BCNW_L, exhibited elongated nanowhiskers with diameters of 67.63 ± 20.1 nm and lengths of 1499.49 ± 25.9 nm, corresponding to solvent-driven swelling and alignment of fibrillar chains. The figure demonstrates that TEM is the ideal tool for structural analysis of BCNWs in TDESs, enabling precise nanowhisker morphology and nanoscale understanding. Table S4 compares the sample length, diameter, and aspect ratio from recently published papers for known fabrication of nanowhiskers.

**Figure 1:**
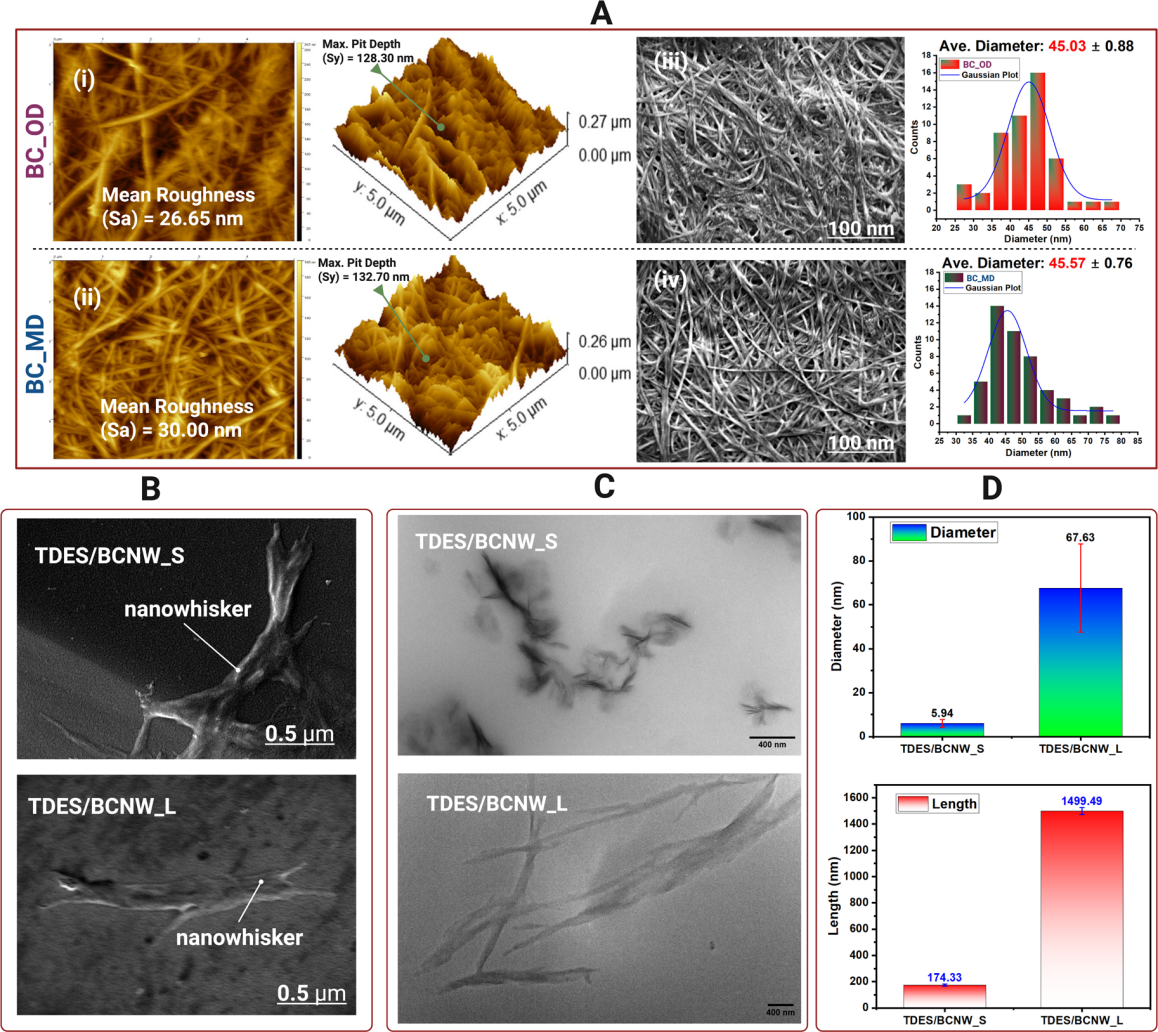
AFM and SEM images (A) of dried bacterial cellulose used before dissolution. SEM images (B) and TEM images (C) of BCNWs after dissolution. Measured BCNW diameter and length using TEM images (D).

Figure 2 shows how nanowhisker structure affects TDES thermal stability, measured by endset temperature and residual percentage. Water-content analysis was excluded because the samples were stored in a desiccator after synthesis. TGA analysis, Figure 2A, showed improved thermal stability upon BCNW inclusion.^11^ The DES has an endset decomposition temperature (*T*_e_) of 308.37 °C, but when tannic acid is added to the mixture, the temperature at which it starts to break down drops significantly to 290.31 °C, probably because of the presence of more unstable hydroxyl-rich groups and greater variety in the mixture.^12^ As expected, when BCNWs are included, a clear reversal takes place: TDES/BCNW_S shows a higher decomposition starting at 348.52 °C, whereas for TDES/BCNW_L, it reaches 336.84 °C. Residual yields (RY) were TDES/BCNW_S = 6.90% and TDES/BCNW_L = 3.53%, while DES and TDES left only 0.45% and 0.63%, respectively, suggesting that BCNWs restrict chain mobility and promote more char formation.^13,14^ DTG profiles, Figure 2B, showed peak degradation temperatures shifting from 276.2 °C for DES and 288.52 °C for TDES to 296.25 and 285.79 °C for TDES/BCNW_S and TDES/BCNMW_L, respectively. This behavior supports the hypothesis that nanowhiskers introduce energy-dissipating surfaces. Table S5 records all thermal degradations observed for all samples wherein thermal decomposition was observed divided into four phases: Phase 1, moisture/volatile loss; Phase 2, organic decomposition; Phase 3, polymer breakdown; Phase 4, carbonization.^15^ Compared to DES, TDES required higher activation energy (*E*_a_), but *E*_a_ dropped in TDES/BCNW systems, indicating controlled degradation, Figure 2C. Entropy (Δ*S*), Figure 2D, and enthalpy (Δ*H*), Figure 2E, became more negative in Phases 3 and 4 for TDES/BCNW systems supporting the notion of BCNWs lowering the randomness of thermal kinetics. In the same phases, the Gibbs free energy (Δ*G*), Figure 2F, increased with BCNWs, confirming the thermodynamic stabilization. Nanowhiskers improved the eutectic matrix by creating networks of hydrogen bonds which led to a stronger and more heat-resistant liquid system.^16^

**Figure 2:**
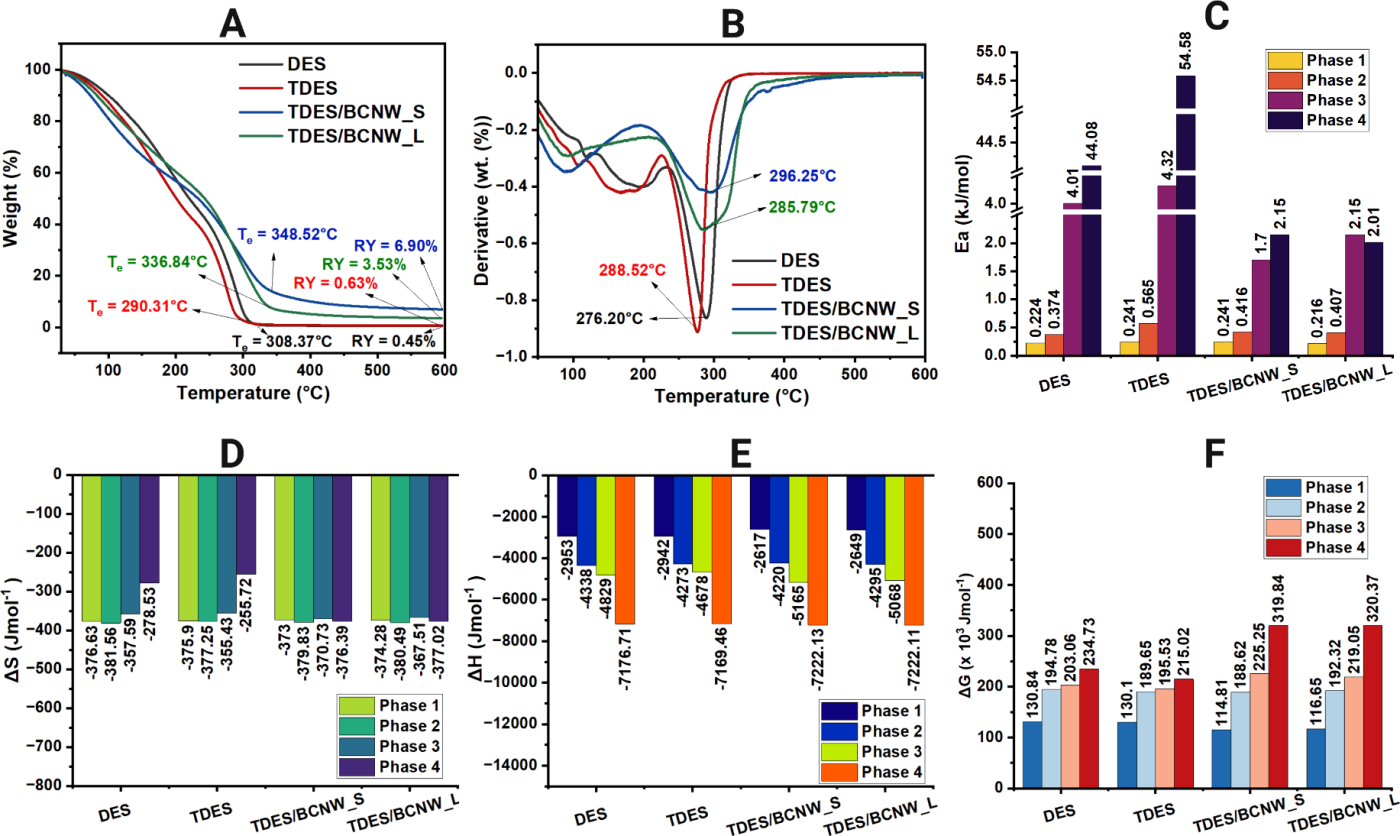
Thermogravimetric and thermodynamic analyses show that adding BCNWs to TDES enhances thermal stability (A), evidenced by higher peak degradation temperatures (B), increased activation energy *E*_a_ (C), and negative Δ*S* (D) and Δ*H* (E). Elevated Gibbs free energy Δ*G* (F) further indicates improved structural order and thermal robustness.

We next assessed how BCNWs influenced TDES physical properties using dynamic light scattering (DLS), polydispersity index (PDI), and zeta potential, see Table 1. DES had a Z-average of 373.8 nm, while TDES reached 751.1 nm due to supramolecular assemblies between imidazole and tannic acid. PDI increased from 0.31 to 0.50 wherein TDES/BCNW_S showed a Z-average of 1364 nm and a high PDI, 0.95, indicating heterogeneity and nanowhisker aggregation, while TDES/BCNW_L showed a lower PDI, 0.32, but a higher Z-average, 6474 nm, suggesting entangled networks of long nanowhiskers, consistent with TEM trends. The size difference between DLS and TEM arises because DLS measures the hydrodynamic diameter of the hydrated state, whereas TEM measures the projected size of individual particles in a dry, drop-cast state. In addition, these structural changes reflect underlying variations in how the nanowhiskers interact with the solvent phase and affect interfacial dynamics, not only dimensions.^17^ Zeta potential analysis revealed major surface charge changes. DES and TDES had negative values (−10.82 and −3.43 mV), due to hydroxyl deprotonation and ionic environment.^18^ With BCNWs addition, the zeta potential became positive: +12.72 mV (TDES/BCNW_S) and +1.96 mV (TDES/BCNW_L). These shifts suggest π–π and hydrogen bonding interactions between tannic acid/imidazole and cellulose and exposure of polar domains on nanowhisker surfaces.^19^

**Table 1:** Dynamic Light Scattering and Zeta Potential Results

Samples	Z-average (nm)	Polydispersity Index (PDI)	Zeta Potential (mV)
DES	373.8 ± 40.4	0.31 ± 0.10	–10.82 ± 1.76
TDES	751.1 ± 52.51	0.50 ± 0.08	–3.43 ± 0.37
TDES/BCNW_S	1364 ± 303.10	0.95 ± 0.01	12.72 ± 0.65
TDES/BCNW_L	6471 ± 165.20	0.32 ± 0.05	1.96 ± 0.11

STEM and EDS analyses, Figure 3, further characterized BCNWs morphology. In TDES/BCNW_S, Figure 3A, nanowhiskers displayed high linearity and twisting, likely due to the TDES disrupting hydrogen bonds. In contrast, TDES/BCNW_L, Figure 3B, showed more curvature and bundling. Twist length and diameter (Figure 3C) increased from 0.36 μm and 22.3 nm in TDES/BCNW_S to 0.60 μm and 52.10 nm in TDES/BCNW_L, respectively, reflecting reduced axial stabilization and enhanced lateral interactions.^20^ Thus, the increased twist length and diameter in TDES/BCNW_L is consistent with greater torsional relaxation.^21^ Elongation ratios near 1.0 (0.937 and 0.914) indicate negligible mechanical scission (Figure 3D).^22^ Full distribution analysis and quantile-based correlations were examined using *Q*–*Q* plots (Figure S1) and summarized in Table S6A,B. A positive Pearson correlation coefficient (PCC) indicated that in TDES/BCNW_S increases in twist length and elongation were accompanied by increases in diameter. In TDES/BCNW_L, twist length and diameter showed a similar positive relationship, whereas twist elongation exhibited an opposite trend with diameter, as reflected by the negative PCC value.^23^ EDS elemental maps, Figure 3(E,F) and Table S7, showed evenly distributed C (84.40%) and O (6.06%) in TDES/BCNW_S with sparse Cl (2.32%) and N (7.22%) while TDES/BCNW_L was composed of C = 79.51%, O = 4.28%, Cl = 5.68%, N = 10.53%, likely due to increased fibril curvature and surface accessibility.^24^

**Figure 3:**
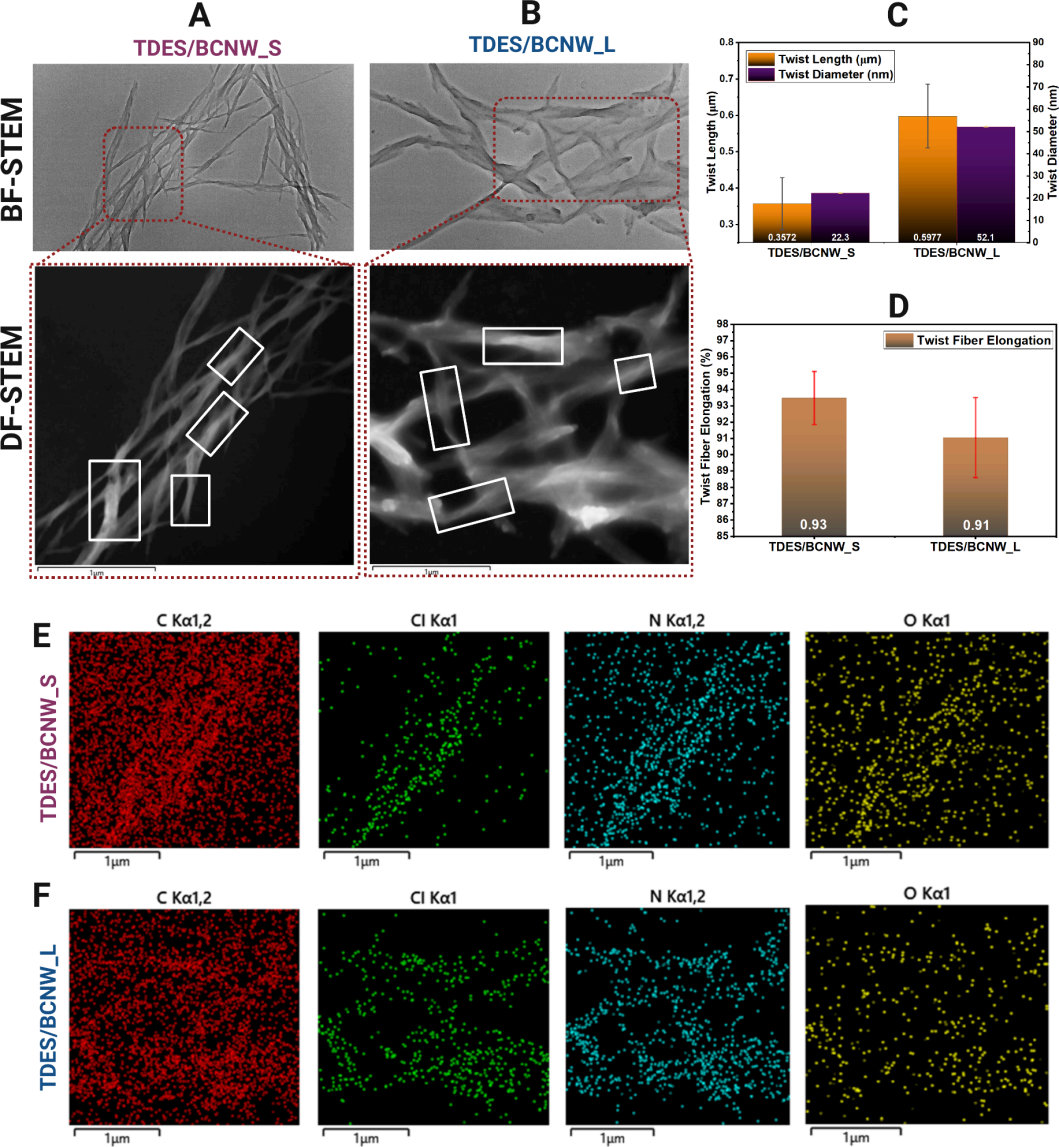
STEM images in Bright Field (BF) and Dark Field (DF) at a 600 nm scale compare BCNWs in TDES/BCNW_S (A) and TDES/BCNW_L (B) showing distinct morphological modulation. Quantitative analysis reveals differences in twist length and diameter (C) and fiber elongation (D). EDS mapping (E, F) highlights variations in C, Cl, N, and O distributions between the two systems.

To assess if BCNWs twist, diameter, and elongation most likely further affect the TDES melting temperature, we used DSC. Broad melting events suggest coexisting solid phases rather than a sharp crystalline phase, aligning with BCNW presence.^25^ In Figure 4A, DES showed sharp peaks wherein the melting point (*T*_m_) started at 419 K and maximum melting temperature (*T*_max_) at 441 K. The presence of tannic acid converted the DES sharp peaks into broad ones resulting in lower *T*_m_ at 406 K and *T*_max_ at 432 K. Further broadening of peaks and lowering of *T*_m_ and *T*_max_ were observed upon the introduction of BCNWs into TDES. This was most likely a result of the increase in viscosity and absorbed stress which means low molecular mobility of the solvent system, see Table S8.^26^

**Figure 4:**
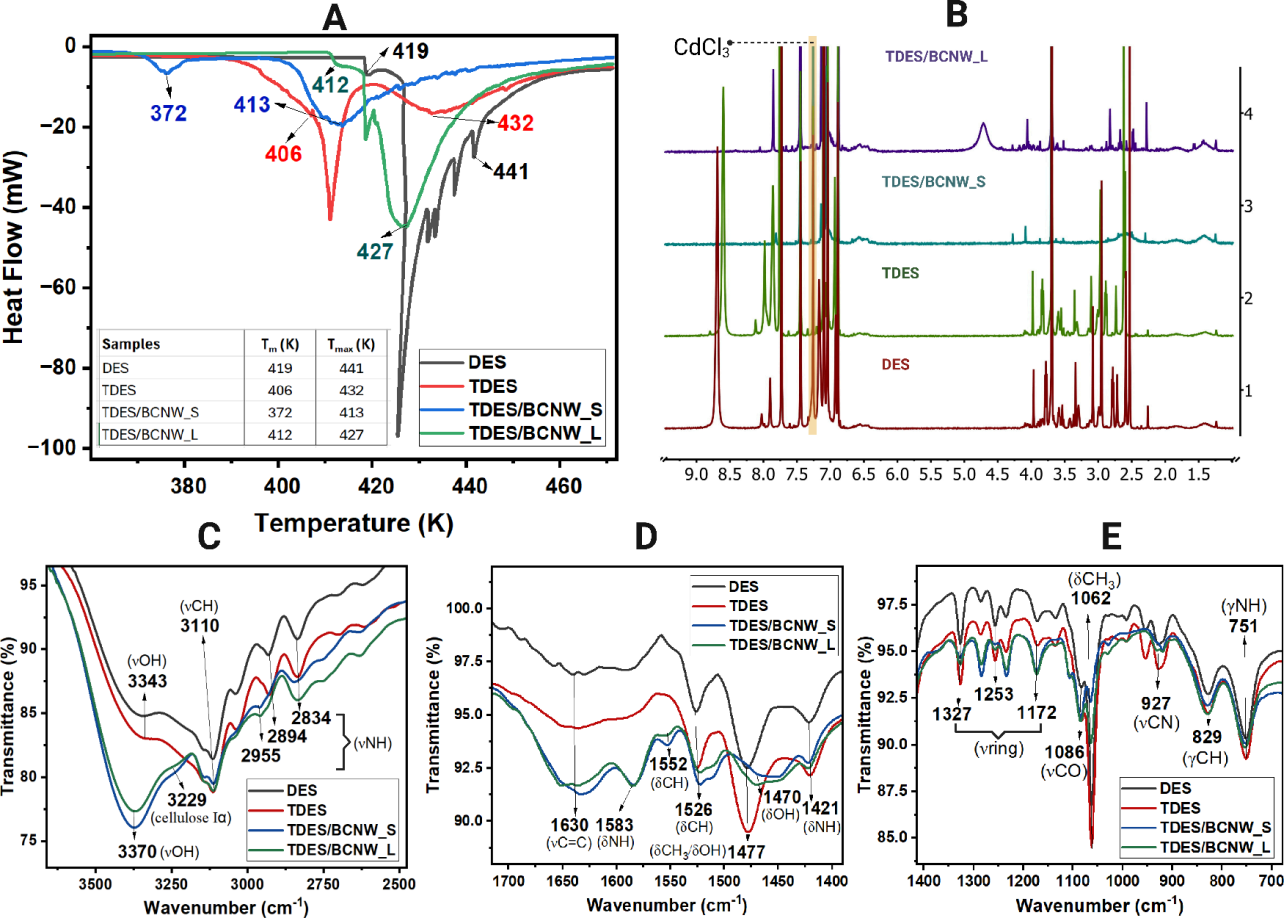
Differential scanning calorimetry (DSC) reveals the distinct melting behaviors of DES, TDES, and TDES/BCNW systems (A). Spectroscopic analysis using ^1^H NMR (B) (ν = stretching, δ = in-plane bending, ρ = rocking, and γ = out-of-plane bending) coupled with ATR-FTIR (C–E).

To investigate intermolecular interactions, we used ATR-FTIR and ^1^H NMR. ^1^H NMR spectra, Figure S2, were assigned based on prior studies.^27–29^ Upon addition of TA and BC, Figure 4B, a new chemical shift at δ 7.6–7.8 ppm in TDES/BCNW systems indicated hydrogen bonding between TA and terminal OH groups in DES.^30^ Signals between δ 2.0 and 3.0 ppm shifted upfield, indicating proton shielding from BC’s hydroxyls interacting with TA’s phenolics. A distinct peak at δ 4.70 ppm for TDES/BCNW_L and δ 2.5 ppm for TDES/BCNW_S confirmed BC nanowhiskers.^29^ Figure S3 shows IR spectra of DES components, choline chloride and imidazole, and their mixture, aligning with Muzio et al.^31^ In the high-frequency region, imidazole exhibited νCH and νNH stretching bands, while ChCl showed a dominant νOH band. Upon mixing at a 3:7 ratio, overlapping of OH and CH bands occurred but the OH mode remained largely unperturbed, indicating continued cation–anion coupling. In the mid- and low-frequency regions, CH (δ, γ) and CH_2_/CH_3_ (γ, ρ) bending modes remained mostly unchanged, with minor shifts in NH (δ, γ) and OH (δ), suggesting NH···OH hydrogen bonding. Figure 4C shows a peak shift from 3343 cm^–1^ to 3370 cm^–1^ after addition of tannic acid and BCNWs, indicating enhanced νOH vibration due to abundant hydroxyls in TA and BC. The 3229 cm^–1^ band, unique to TDES/BCNW_S and _L, signifies the presence of BC, consistent with its Iα structure.^32,33^ An increase in % transmittance at 3110 cm^–1^ (νCH) and a νNH shift from 2894 to 2955 cm^–1^ confirm the BC presence and NH···OH interactions. New peaks at 1552 cm^–1^ (δCH) and 1526 cm^–1^ (δNH), broadening at 1470 cm^–1^ (δOH), and intensification at 1630 cm^–1^ (νC=C) indicated the presence of more aromatic rings from tannic acid, which strongly induces hydrogen bonding to BC, Figure 4D. In Figure 4E, the fingerprint region shows reduced transmittance at 1062 cm^–1^ (δCH_3_ from ChCl) and intensified peaks at 1172 cm^–1^ (νring from imidazole, TA, and BC), reflecting nonaromatic ring effects. Slight reductions at 927 cm^–1^ (νCN) and 751 cm^–1^ (γNH) in TDES/BCNW_S and _L confirm strong OH···OH interactions between TA and BC.

Overall, TEM showed that bacterial cellulose in TDES restructures into nanowhiskers with varied dimensions, enhancing thermal stability and melting temperature and altering zeta potential along with particle size, evidencing interfacial interactions. This BCNW–TDES integration provides a green, tunable platform for advanced materials and potential polymer electrolyte applications.^34^

## Supplementary Material



## Notes

Experimental section (materials, culturing and fabrication of bacterial nanocellulose, design of experiment and synthesis of deep eutectic solvent and dissolution of bacterial cellulose); Reaction conditions and parameters of bacterial cellulose dissolution in TDES; Characterizations; Nanowhisker diameter and length from TEM analysis of samples miscible in water; Weighted masses used for bacterial nanocellulose dissolution in choline chloride–imidazole–tannic acid deep eutectic solvent; Comparison of BCNW Dimensions and solvent used to other existing known nanowhiskers; Thermal degradation and residual yield results; Correlation of Twist Length vs Twist Diameter vs Elongation for TDES/BCNW_S and TDES/BCNW_L; Chemical composition using TEM-EDS; Viscosity and absorbed stress; *Q*–*Q* plot for samples; ^1^H NMR spectra of DES components; and FTIR spectra of DES components (PDF)
