# Supplementary material for: Transmission Electron Microscopy for Structural Insights into Bacterial Cellulose Nanowhiskers in Ternary Deep Eutectic Solvent
Source: ACS Meas Sci Au. 2026 Jan 2;6(1):28–34. doi: 10.1021/acsmeasuresciau.5c00159 (PMC12921604; doi:10.1021/acsmeasuresciau.5c00159)
Supplement: Supplementary file 1 [file tg5c00159_si_001.pdf]

Supporting Information

Transmission Electron Microscopy for Structural Insights of Bacterial Cellulose Nanowhiskers in Ternary Deep Eutectic Solvent

[1]Maurelio Cabo Jr., [1]Abed Alqader Ibrahim, [1]Gayani Pathiraja, [2]Farbod Ebrahimi, [2]Shobha Mantripragada, [1]Omiya Ayoub, [1]Besan Khader, [2]Kristen Dellinger, [2]Jeffrey R. Alston, [1]Sherine O. Obare, [1]*Dennis R. LaJeunesse

[1]Department of Nanoscience, Joint School of Nanoscience and Nanoengineering, The University of North Carolina at Greensboro, NC, USA, 27401
[2]Department of Nanoengineering, Joint School of Nanoscience and Nanoengineering, North Carolina Agricultural and Technical State University, NC, USA, 27402

Corresponding Author: drlajeun@uncg.edu*

A. Experimental Section

A.1. Materials

Dextrose, Yeast extract, NaOH pellets, Na_2HPO_4, Agar powder, Peptone, Tannic Acid, Choline Chloride (99%), and Ethanol 70% were purchased from Fisher Scientific (Thermo Fisher Scientific, Waltham, MA, USA). Imidazole (99%) and Citric Acid were purchased from Sigma-Aldrich (Sigma-Aldrich, St. Louis, MO, United States). Pellicles of Bacterial Cellulose (BC) were collected from cultures of *Gluconacetobacter hansenii* (ATCC 23769, American Type Culture Collection, Manassas, VA, USA).

A.2. Culturing and Fabrication of Bacterial Cellulose

Gluconacetobacter hansenii was cultured in Hestrin-Schramm (HS) media composed of 2% (w/v) Dextrose, 0.5% (w/v) yeast extract, 0.5% (w/v) peptone, 0.27% (w/v) Na_2HPO_4, and 0.125% (w/v) citric acid. To inoculate the bacterial strain, an agar plate (85 mm × 15 mm) with the same HS media composition and 2% agar was prepared and incubated at 30°C for 2 days [1]. A pea-sized bacterial colony was then transferred from the inoculation plate into 1000 mL of media in a 9” × 13” (3-quart) Anchor Hocking glass baking dish and cultured for 14 days at 25°C. After the incubation period, the pellicles were treated in a selected NaOH concentration at 95°C for 1 hour to remove bacterial cells and biofilm materials, leaving only the bacterial nanocellulose matrix. The pellicles were then washed with distilled water until a neutral pH was reached and stored in deionized (DI) water at room temperature.

A. 3. Design of Experiment

From previously established methods and dissolution conditions and parameters [2], we replicated this wherein as shown in Table S1.

Table S1: Reaction conditions and parameters of bacterial cellulose dissolution in TDES				
Sample Name	Pre-Dissolution		During Dissolution	
	Drying Technique (A)	Alkaline Treatment Concentration (B)	%w/v (C)	Set Temperature (D)
E1	*OD	0.3 M	1.5%	180ºC
E2	**FD	0.1 M	1.0%	180ºC
E3	FD	0.3 M	0.5%	160ºC
E4	***MD	0.1 M	1.5%	160ºC
E5	MD	0.2 M	0.5%	180ºC
*OD = Oven Drying; **FD = Freeze-Drying; ***MD = Microwave Drying				

A.4 Synthesis of Deep Eutectic Solvent and Dissolution of Bacterial cellulose

In 3:7 molar ratio [3], we mixed choline chloride (8.39 g) and imidazole (9.53 g) and heat up at 120ºC in a hotplate for 3 hours. The mixture turned into yellowish liquid solution and let it rest for 24 h in room temperature. An amount of tannic acid, 0.5% of the total volume retrieved from DES, was added in the following day and the liquid color transformed into a reddish black shade. Weighted masses used to solubilize bacterial nanocellulose were measured from 0.5% to 1.5% of the total volume retrieved from TDES [2]. After dissolution into the TDES solution the color of the mixture changed into darkened solution phase. Table S2 disclosed the amount used per component.

Table S2: Weighted masses used for Bacterial cellulose dissolution in Choline Chloride/Imidazole/Tannic Acid Deep Eutectic Solvent				
Sample Name	Choline Chloride (g)	Imidazole (g)	Weight of Tannic Acid Added (mg)	Weight of BNC Solubilized (mg)
E1	8.39	9.53	86.7	202.7
E2			86.8	138.5
E3			76.7	63.30
E4			76.9	204.30
E5			82.8	65.60

B. Characterizations

Before dissolution, all alkaline-treated bacterial nanocellulose was characterized using SEM and AFM. The scanning electron micrographs were obtained using a JEOL JSM-IT800 Schottky FESEM (Zeiss, Jena, Germany). Nanowhisker diameters were measured using ImageJ software (U.S. National Institutes of Health, Bethesda, Maryland, USA), 50 times per sample. The surface details, like how rough it is and how deep the pits are on dried bacterial nanocellulose, were measured using an Asylum-3D Origin + AFM in a non-contact way with Tap-150G AFM probes from Ted Pella, which have specific sizes and strengths (m). We conducted the measurements at ambient temperatures and processed the images using Gwyddion software packages.

The morphology and elemental mapping of bacterial cellulose samples were investigated using JEOL 2100PLUS high-resolution transmission electron microscopy (HR-TEM) coupled with a STEM/EDS capability operated at an accelerating voltage of 200 kV. To prepare the sample, 10 µL was mixed with 600 µL of 70% ethanol in Eppendorf tubes (0.6 mL), 1:100 serial dilution, then it was sonicated for 30 minutes set at room temperature and placed on a carbon-coated copper TEM grid for analysis. Nanowhisker diameter, length, and twist were also measured using ImageJ software (U.S. National Institutes of Health, Bethesda, Maryland, USA), 25 times per sample for length and diameter, and 10 times per sample for twist. Fiber elongation [4] and aspect ratio [5] were calculated based on the following equations:

Elongation = 1-(d/ l) *(1)*

Aspect Ratio = (l/d) (2)

where d = fiber diameter and l = fiber length. The fiber twist was measured from the points where the ribbon begins to narrow around the twisted region [6]. All measurements were performed on images acquired under the original scale (1µm) and no adjustment in contrast settings to ensure consistency.

Q-Q plot was employed using OriginPro software to study the full distribution and correlation of twist length, elongation and diameter [7]. An additional statistical analysis using Pearson Correlation Coefficient (PCC) [8] was used to further assess the correlation between length vs diameter and diameter vs elongation using the following equations:

Mean: $\mu = \frac{1}{n}\sum x_i$ (3)

wherein n = no. of samples and $\sum x_i$ = sum of all the samples.

Covariance = $cov\ (x, y) = \frac{1}{n-1}\sum(x_i - \mu)(y_i - \mu)$ (4)

wherein n = no. of samples and $\sum(x_i - \mu)(y_i - \mu)$ = the product of the sum of two comparing samples.

Standard deviations: $\sigma_x = \sqrt{\frac{\sum(x_i-\mu)^2}{n-1}}$ (5)

Pearson Correlation Coefficient: $r = \frac{cov\ (x,y)}{\sigma_x \sigma_y}$ (6)

IR spectra were recorded on a Fourier-transform infrared spectroscopy (FTIR) spectrometer (Agilent 670 FTIR Spectrometer, Santa Clara, CA, USA) under dry air at ambient temperature. The percentage of transmittance spectra was recorded from 4000 to 400 cm^{-1} with 64 scans in each case at a resolution of 4 cm^{-1}. 1H-NMR spectra were recorded using Agilent 400NMR.

The size and zeta potential measurements were performed using a Malvern Zetasizer Ultra instrument (Malvern Panalytical, UK) at 25°C. Samples were diluted to an appropriate concentration, 10 µL was mixed with 600 µL of 70% ethanol and analyzed in folded capillary cells (DTS1070, Malvern Panalytical). The hydrodynamic diameter, polydispersity index (PDI), zeta potential, and conductivity were measured three times, and the results are shown as an average with the variation included.

The thermal stabilities of DES, TDES, and TDES/BCNW solvent systems were characterized using a thermogravimetric analyzer (TGA; Perkin Elmer STA 6000, England) within a temperature range of 30 to 600°C at a rate of 20°C/min under an air atmosphere. The kinetic parameters were determined using a modified form of the Coats and Redfern model as described [9-10] in the following equations:

$$ln\,[-\,ln(1-x)] = ln\,(\frac{ART^2}{\beta E_a} - \frac{E_a}{RT}) \qquad (7)$$

where x is the first rate if reaction, A is the pre-exponential factor, β is the heating rate (20 °C/min), R is the general gas constant (8.3143 $Jmol^{-1}\,K^{-1}$), E_a is the activation energy, and T is the temperature (K). Plotting graphs between ln[−ln(1 − x)] vs 1000/T for each phase gave the value of activation energy, and further parameters were determined using basic thermodynamic equations [11] .

Entropy change: $$\Delta S = R\left[ln\left(\frac{Ah}{KT}\right)\right] \qquad (8)$$

where h is the Planck constant and K is the Boltzmann constant.

Enthalpy change: $$\Delta H = E_a - RT \qquad (9)$$

Gibbs free energy change: $$\Delta G = \Delta H - T\Delta S \qquad (10)$$

To avoid potential water absorption, samples were stored in a desiccator after each synthesis. The melting temperatures were measured via DSC technique. Samples were weighed into aluminum pans (10-20 mg), sealed, and analyzed with modulated runs in the temperature range from −60 to 200°C, with an isothermal run over the first 2 mins at 50°C and then with a heating rate of 5°C/min in a nitrogen atmosphere and a second isothermal run in 15 mins at 15°C. In graph analysis, we converted the temperature into Kelvin by adding 273 into Celsius degree to align with related studies. For viscosity and abosrbed stress were emasured using TA HR20 Rheometer.

Table S3: Nanowhiskers Diameter and Length from TEM Analysis of Samples

Selected Samples	Length (nm)	Diameter (nm)	Aspect ratio
E1	174.33 ± 8.40	5.94 ± 1.84	29.35
E2	1052.63 ± 17.31	54.62 ± 13.32	19.27
E3	384.23 ± 22.29	8.70 ± 3.04	44.16
E4	862.95 ± 49.12	60.29 ± 19.47	14.31
E5	1499.49 ± 25.88	67.63 ± 20.10	22.17

Note: E1 and E5 were renamed into TDES/BCNW_S and TDES/BCNMW_L, respectively.

Table S4. Comparison of BCNW Dimensions and Solvent used to other existing known Nanowhiskers					
Source	Solvent Used	Length (nm)	Diameter (nm)	Aspect Ratio	Reference(s)
Bacterial cellulose	TDES	174.33 to 1499.49	5.94 to 67.63	14.31 to 44.16	This study
Microcrystalline Cellulose (MCC)	Sulfuric Acid	199 to 344	11 to 33	10 to 18	[12]
Fiber Arrowroot	Sulfuric Acid	121 to 160	3.1 to 4.1	33 to 46	[13]
Cotton Linter	Sulfuric Acid	250	10	25	[14]
Sisal	TEMPO/NaBr/NaClO	500 to 600	5 to 10	60 to 100	[15]
Jute	TEMPO/NaBr/NaClO	100 to 200	3 to 10	20 to 33	[16]
Curaua	TEMPO/NaBr/NaClO	102	12	8.5	[17]
Microcrystalline Cellulose (MCC)	TEMPO/NaBr/NaClO	200 to 400	5 to 10	20 to 80	[18]
Purified Cellulose Powder	Hydrobromic Acid	180	6	30	[19]
Softwood Kraft Pulp	Sulfuric Acid	150 to 300	4 to 8	18 to 75	[20]
Cotton	Sulfuric Acid	100 to 40,000	4 to 20	-	[21]

Table S5: Thermal degradation and residual yield results							
Samples	$^{a}T_o$ (°C)	$^{b}T_m$ (°C)	$^{c}T_p$ (°C)	$^{d}T_e$ (°C)	$^{e}W_{L-1}$ (%)	$^{f}W_{L-2}$ (%)	^{g}RY (%)
DES	82.24	248.85	276.20	308.37	54.15	45.40	0.45
TDES	80.94	241.05	288.52	290.31	57.11	42.26	0.63
TDES/BCNW_S	41.83	234.69	296.25	348.53	46.37	46.73	6.90
TDES/BCNW_L	45.78	243.75	285.79	336.84	46.16	50.31	3.53

a – onset Temperature
b – midpoint Temperature (transition)
c – peak Temperature measured from Derivative weight
d – endset temperature
e – weight loss for 1st degradation
f – weight loss for 2nd degradation
g – residual yield

SN #	Table S6-A: Correlation of Twist Length vs Twist Diameter vs Elongation for TDES/BCNW_S										
	Length (L) (µm)	Diameter (D) (µm)	Elongation (E)	L_i - L_m	D_i - D_m	E_i - E_m	Covariance (L and D)	Covariance (D and E)	(L_i - L_m)* (L_i - L_m)	(D_i - D_m)* (D_i - D_m)	(E_i - E_m)* (E_i - E_m)
1	0.271	0.017	0.937269373	-0.0862	-0.0053	0.000604142	0.00045686	-3.20195E-06	0.00743044	0.00002809	3.64987E-07
2	0.275	0.019	0.930909091	-0.0822	-0.0033	-0.00575614	0.00027126	1.89953E-05	0.00675684	0.00001089	3.31331E-05
3	0.293	0.019	0.935153584	-0.0642	-0.0033	-0.001511647	0.00021186	4.98844E-06	0.00412164	0.00001089	2.28508E-06
4	0.312	0.022	0.929487179	-0.0452	-0.0003	-0.007178051	1.356E-05	2.15342E-06	0.00204304	9E-08	5.15244E-05
5	0.314	0.022	0.929936306	-0.0432	-0.0003	-0.006728925	1.296E-05	2.01868E-06	0.00186624	9E-08	4.52784E-05
6	0.348	0.022	0.936781609	-0.0092	-0.0003	0.000116378	2.76E-06	-3.49135E-08	8.464E-05	9E-08	1.35439E-08
7	0.382	0.024	0.937172775	0.0248	0.0017	0.000507544	0.00004216	8.62825E-07	0.00061504	2.89E-06	2.57601E-07
8	0.427	0.024	0.943793911	0.0698	0.0017	0.00712868	0.00011866	1.21188E-05	0.00487204	2.89E-06	5.08181E-05
9	0.446	0.026	0.941704036	0.0888	0.0037	0.005038805	0.00032856	1.86436E-05	0.00788544	0.00001369	2.53896E-05
10	0.504	0.028	0.944444444	0.1468	0.0057	0.007779214	0.00083676	4.43415E-05	0.02155024	0.00003249	6.05162E-05
Mean	0.3572	0.0223	0.936665231			Sum	0.0022954	0.000100886	0.0572256	0.0001021	0.000269581
						Covariance	0.000255044	1.12095E-05			

Standard Deviation (L) 0.0063584

0.079739576

Pearson Correlation Coefficient (PCC) 0.949621041 L vs D

R^2 = 0.901780122

Standard Deviation (D) 1.13444E-05

0.003368151

Pearson Correlation Coefficient (PCC) 0.608095334 D vs E

Standard Deviation (E) 2.99534E-05

0.005472974

R^2= 0.369779936

Table S6-B: Correlation of Twist Length vs Twist Diameter vs Elongation for TDES/BCNW_L

SN #	Length (μm)	Diameter (μm)	Elongation (E)	$L_i - L_m$	$D_i - D_m$	$E_i - E_m$	Covariance (L and D)	Covariance (D and E)	$(L_i - L_m)*(L_i - L_m)$	$(D_i - D_m)*(D_i - D_m)$	$(E_i - E_m)*(E_i - E_m)$
1	0.453	0.034	0.924944812	-0.1447	-0.0181	0.011416186	0.00261907	-0.000206633	0.02093809	0.00032761	0.000130329
2	0.51	0.045	0.911764706	-0.0877	-0.0071	-0.00176392	0.00062267	1.25238E-05	0.00769129	5.041E-05	3.11142E-06
3	0.549	0.045	0.918032787	-0.0487	-0.0071	0.004504161	0.00034577	-3.19795E-05	0.00237169	5.041E-05	2.02875E-05
4	0.559	0.048	0.914132379	-0.0387	-0.0041	0.000603753	0.00015867	-2.47539E-06	0.00149769	1.681E-05	3.64518E-07
5	0.56	0.048	0.914285714	-0.0377	-0.0041	0.000757088	0.00015457	-3.10406E-06	0.00142129	1.681E-05	5.73182E-07
6	0.6	0.053	0.911666667	0.0023	0.0009	-0.00186196	2.07E-06	-1.67576E-06	5.29E-06	8.1E-07	3.46689E-06
7	0.602	0.054	0.910299003	0.0043	0.0019	-0.003229623	8.17E-06	-6.13628E-06	1.849E-05	3.61E-06	1.04305E-05
8	0.664	0.056	0.915662651	0.0663	0.0039	0.002134024	0.00025857	8.32269E-06	0.00439569	1.521E-05	4.55406E-06
9	0.685	0.059	0.913868613	0.0873	0.0069	0.000339987	0.00060237	2.34591E-06	0.00762129	0.00004761	1.15591E-07
10	0.795	0.079	0.900628931	0.1973	0.0269	-0.012899696	0.00530737	-0.000347002	0.03892729	0.00072361	0.000166402
Mean	0.5977	0.0521	0.913528626			Sum	0.0100793	-0.000575813	0.0848881	0.0012529	0.000339635
						Covariance	0.001119922	-6.39793E-05			

Standard Deviation (L): 0.009432011; 0.097118542

Pearson Correlation Coefficient (PCC): 0.977346947 — L vs D

R^2 = 0.955207054

Standard Deviation (D): 0.000139211; 0.011798776

Pearson Correlation Coefficient (PCC): -0.882708484 — D vs E

R^2 = 0.779174267

Standard Deviation (E): 3.77372E-05; 0.006143063

Table S7: Chemical Composition using TEM-EDS								
Samples	Weight %				Atomic Weight %			
	C	N	O	Cl	C	N	O	Cl
TDES/BNC_S	84.40 ± 0.80	7.22 ± 0.68	6.06 ± 0.42	2.32 ± 0.21	87.99	6.45	4.74	0.82
TDES/BNC_L	79.51 ± 1.03	10.53 ± 0.89	4.28 ± 0.44	5.68 ± 0.38	84.87	9.64	3.43	2.06

Table S8: Viscosity and Absorbed Stress		
Samples	Viscosity (Pa.s)	Absorbed Stress (dyne/cm²)
DES	0.131 ± 0.09	12.21 ± 0.52
TDES	0.132 ± 0.09	12.39 ± 0.50
TDES/BCNW_S	0.248 ± 0.12	25.41 ± 3.23
TDES/BCNW_L	0.174 ± 0.10	17.18 ± 1.36

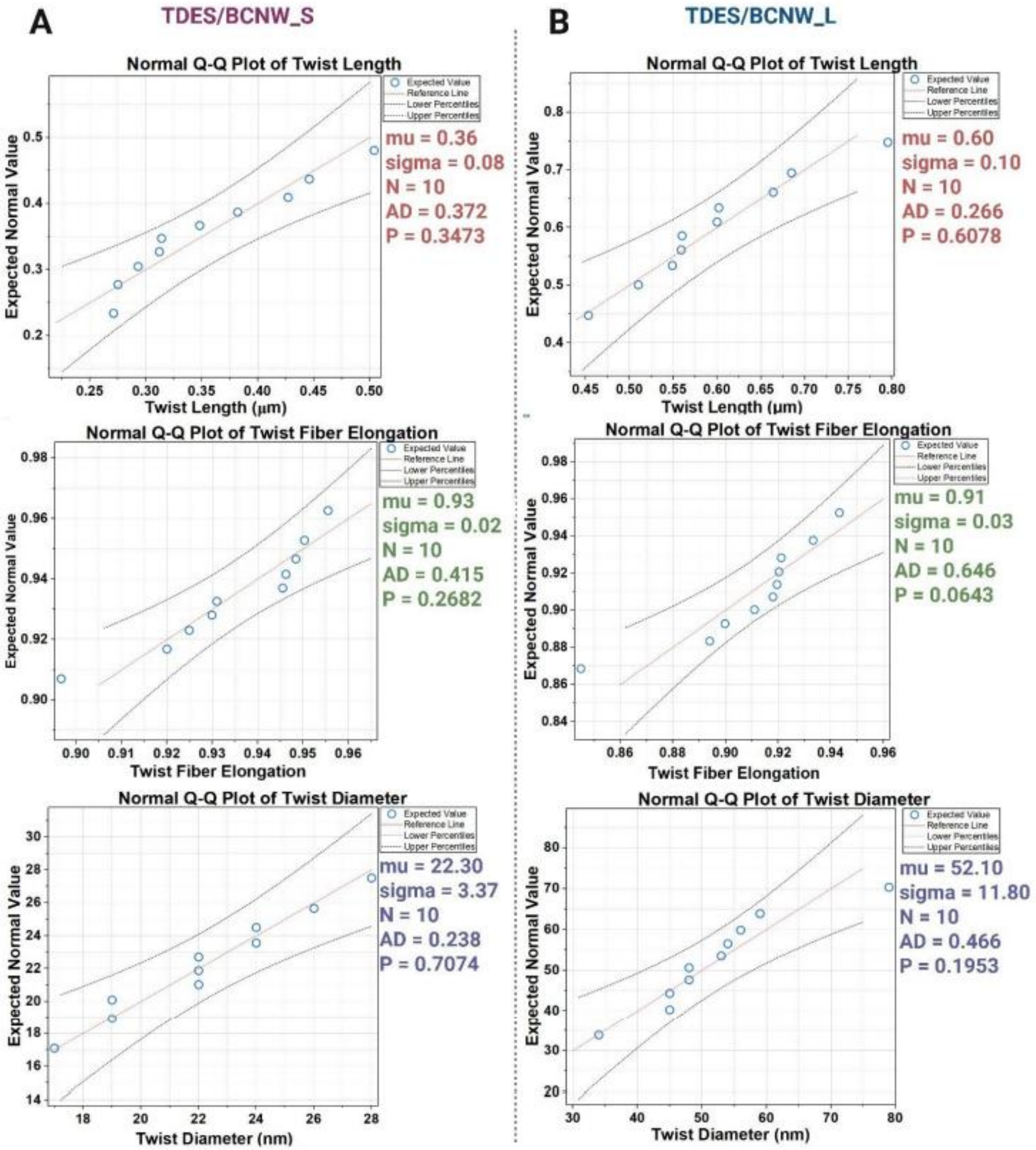

Figure S1: Q-Q Plot of twist length, diameter and fiber elongation for (A) TDES/BCNW_S; and (B) TDES/BCNW_L

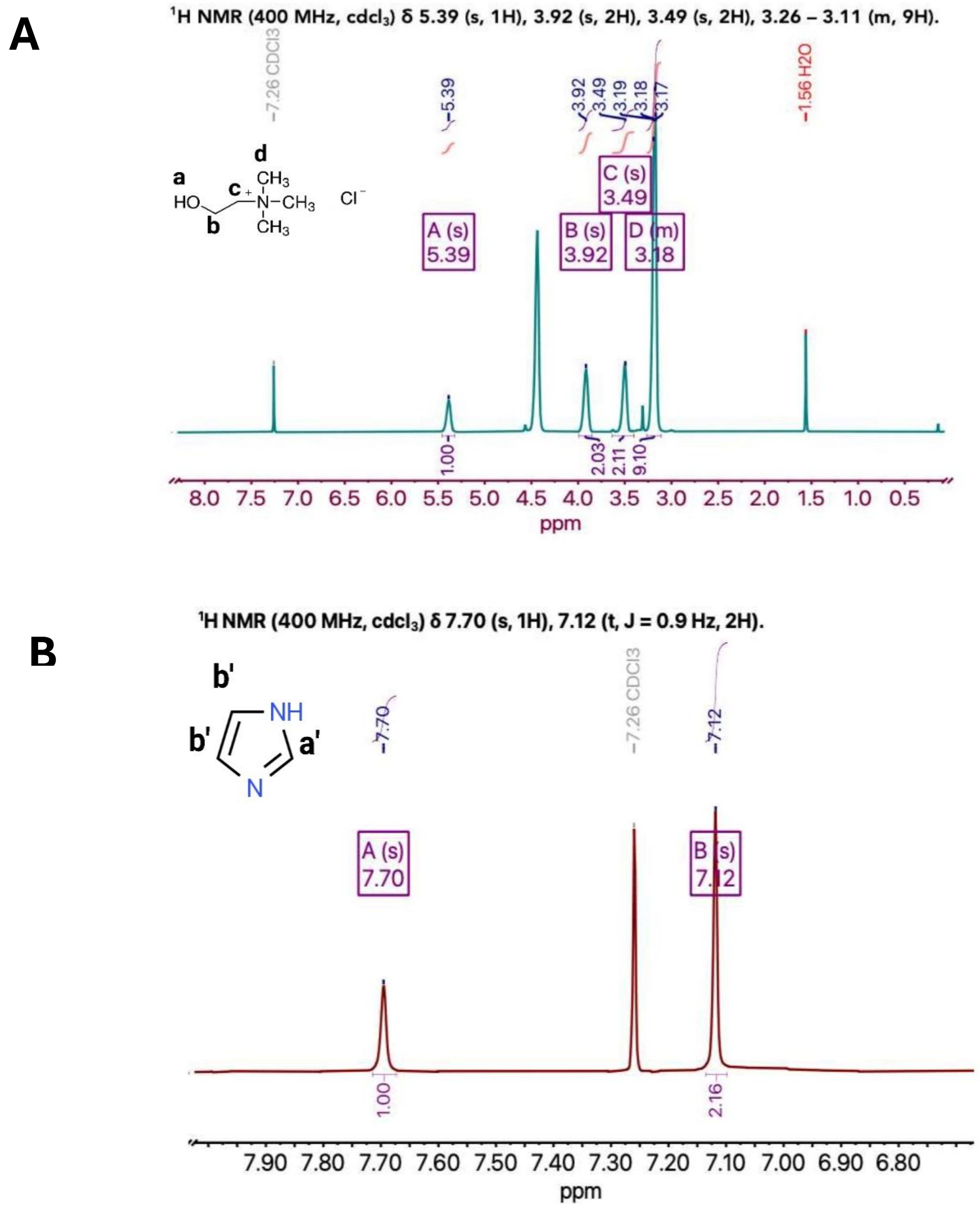

Figure S2: ^{1}H-NMR spectra of DES components: Choline Chloride (A) and Imidazole (B). We assigned the following proton chemical shifts for DES as follows: For choline chloride— ^{1}H NMR (400 MHz, cdcl$_3$) δ 5.39 (s, 1H), 3.92 (s, 2H), 3.49 (s, 2H), 3.26 – 3.11 (m, 9H) and for imidazole, the shifts ^{1}H NMR (400 MHz, cdcl$_3$) δ 7.70 (s, 1H), 7.12 (t, J = 0.9 Hz, 2H).

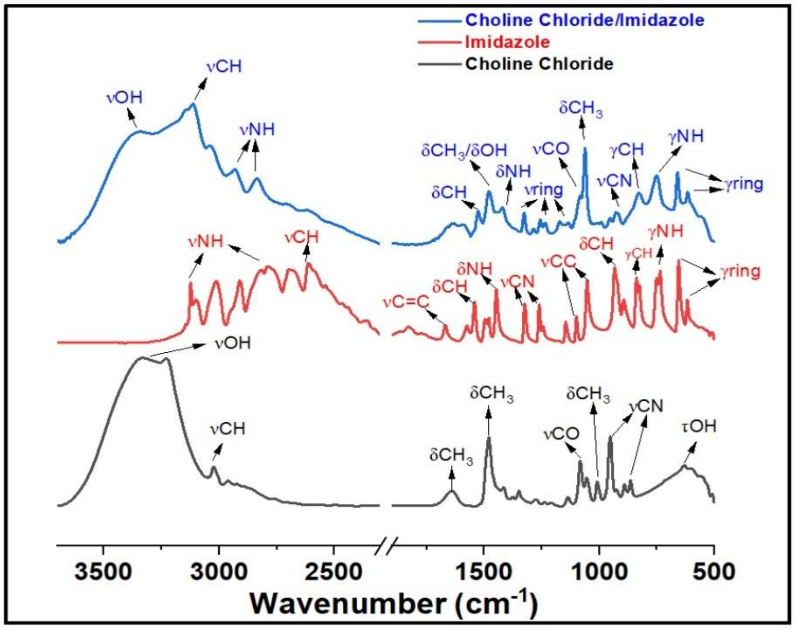

Figure S3: FTIR spectra of DES components

References:

1. Cabo, M. More, N. Alston, J.R. *et al.* "Insight on the Mechanical Properties of Facile Hydrophobic-Barrier-Patterned Bacterial Nanocellulose via Self-Bonding Mechanism," *ACS Nanoscience Au*, 5, 3(2025): 128-136. https://doi.org/10.1021/acsnanoscienceau.4c00077
2. Cabo, M.Jr.; Kattel, S.; LaJeunesse, D. Tuning Ternary Deep Eutectic Solvent Semiconductivity and Specific Capacitance Properties via Solubilizing Bacterial Nanocellulose for Flexible Soft Material. ACS Materials Au. 2025, 5, 971-977.
3. Kian, L.K. Jawaid, M. "Thermal Properties of Nanocrystalline Cellulose and Cellulose Nanowhisker," *IJITEE*, 9, 1 (2019): 5430-5434. https://doi.org/10.35940/ijitee.A8103.119119
4. MEASURING THE LENGTH AND WIDTH OF FIBERS ON THE MORPHOLOGI G3. Link: https://www.atascientific.com.au/wp-content/uploads/2017/02/MRK1918-01_Measuring_length_width_fibers_w-MorpholG3-.pdf. Accessed on June 27, 2025.
5. Fiber Reinforcements. Link: https://www.addcomposites.com/post/fiber-reinforcements. Accessed on June 27, 2025.
6. Babi, Mouhanad,; Williams, A.; Reid, M.; Grandfield, K.; Bassim, N.D.; Moran-Mirabal, J.M. "Unraveling the Supramolecular Structure and Nanoscale Dislocations of Bacterial Cellulose Ribbons Using Correlative Super-Resolution Light and Electron Microscopy," Biomacromolecules, 2023, 24, 258-268.
7. OriginLab. Re: 17.2.4 Probability Plot and Q-Q Plot. Link: https://www.originlab.com/doc/en/Origin-Help/ProbPlot-QQPlot. Accessed: Dec. 3, 2025
8. University of Virginia Library. Re: Correlation: Pearson, Spearman, and Kendall's tau. Link: https://library.virginia.edu/data/articles/correlation-pearson-spearman-and-kendalls-tau. Accessed: Dec. 3, 2025.
9. Cabo, M. Prabhakar, M.N. Lee, D.W. Yu, R. Chanthavong, V. Song, J.I. "Improving the Flame Retardancy and Mechanical Properties of Vinyl Ester Resins through Maleated Epoxidized Corn

Oil/Epoxy Resin Additives for Sustainable Thermoset Composites," *ACS Polym. Au*, 5, 1 (2025): 45-58. https://doi.org/10.1021/acspolymersau.4c00088

10. Farrukh, M. A. Butt, K. M. Chong, K. Chang, W. S. "Photoluminescence emission behavior on the reduced band gap of Fe doping in CeO2-SiO2 nanocomposite and photophysical properties," *J. Saudi Chem. Soc.,* 23, 5 (2019): 561−575. https://doi.org/10.1016/j.jscs.2018.10.002
11. Chen, C. Bu, X. Huang, D. Huang, Y.; Huang, H. "Thermal Decomposition and Kinetics Analysis of Microwave Pyrolysis of Dunaliella salina Using Composite Additives," *Bioenergy Res.* 13, 4 (2020): 1205−1220. https://doi.org/10.1007/s12155-020-10150-7
12. Pan, M., Zhou, X., and Chen, M. (2013). "Cellulose nanowhiskers isolation and properties from acid hydrolysis combined with high pressure homogenization," *BioRes.* 8(1), 933-943. https://dx.doi.org/10.15376/biores.8.1.933-943
13. Sá, R.M.D. Miranda, C.S. José, N.M. "Preparation and Characterization of Nanowhiskers Cellulose from Fiber Arrowroot (Maranta arundinacea)," Mat. Res., 18, 2 (2015): 225-229. http://dx.doi.org/10.1590/1516-1439.366214
14. Ni, H. Zeng, S. Wu, J. et al. "Cellulose nanowhiskers: Preparation, characterization and cytotoxicity evaluation," Bio-Medical Materials and Engineering, 22, 1-3(2012): 121-127. https://dx.doi.org/10.3233/bme-2012-0697
15. Fan, F. Zhu, M. Fang, K. et al. "Extraction and characterization of cellulose nanowhiskers from TEMPO oxidized sisal fibers," Cellulose, 29, 1(2022): 213-222. https://doi.org/10.1007/s10570-021-04305-8
16. Cao, X. Ding, B. Yu, J. Al-Deyab, S.S. "Cellulose nanowhiskers extracted from TEMPO-oxidized jute fibers," Carbohydrate Polymers, 90, 2(2012): 1075-1080. https://doi.org/10.1016/j.carbpol.2012.06.046
17. Motta Neves, R. Silveira Lopes, K. Zimmermann, M. Poletto, M. Zattera, A.J. "Cellulose Nanowhiskers Extracted from Tempo-Oxidized Curaua Fibers," Journal of Natural Fibers, 17, 9(2020): 1355-1365. https://doi.org/10.1080/15440478.2019.1568346
18. Ma, H. Burger, C. Hsiao, B.S. Chu, B. "Nanofibrous Microfiltration Membrane Based on Cellulose Nanowhiskers,' Biomacromolecules, 13, 1(2012): 180-186. https://doi.org/10.1021/bm201421g
19. Mashkour, M. Kimura, T. Kimura, F. Mashkour, M. Tajvidi, M. "Tunable Self-Assembly of Cellulose Nanowhiskers and Polyvinyl Alcohol Chains Induced by Surface Tension Torque," Biomacromolecules, 15, 1(2014): 60-65. https://doi.org/10.1021/bm401287s
20. Dash, R. Ragauskas, A.J. "Synthesis of a novel cellulose nanowhisker-based drug delivery system," RSC Adv. 2, 8(2012): 3403. https://doi.org/10.1039/C2RA01071B
21. Thielemans, W. Warbey, C.R. Walsh, D.A. "Permselective nanostructured membranes based on cellulose nanowhiskers," Green Chemistry, 11, 4(2009): 531. https://doi.org/10.1039/B818056C
